# Gait kinematic analysis in patients with a mild form of central cord syndrome

**DOI:** 10.1186/1743-0003-8-7

**Published:** 2011-02-02

**Authors:** Angel Gil-Agudo, Soraya Pérez-Nombela, Arturo Forner-Cordero, Enrique Pérez-Rizo, Beatriz Crespo-Ruiz, Antonio del Ama-Espinosa

**Affiliations:** 1 Biomechanics and Technical Aids Unit, Department of Physical Medicine and Rehabilitation, National Hospital for Spinal Cord Injury. SESCAM. Finca the Peraleda s/n, Toledo, 45071, Spain; 2 Biomechatronics Laboratory, Mechatronics Department, Polytechnic School of the University of São Paulo, Brazil

## Abstract

**Background:**

Central cord syndrome (CCS) is considered the most common incomplete spinal cord injury (SCI). Independent ambulation was achieved in 87-97% in young patients with CCS but no gait analysis studies have been reported before in such pathology. The aim of this study was to analyze the gait characteristics of subjects with CCS and to compare the findings with a healthy age, sex and anthropomorphically matched control group (CG), walking both at a self-selected speed and at the same speed.

**Methods:**

Twelve CCS patients and a CG of twenty subjects were analyzed. Kinematic data were obtained using a three-dimensional motion analysis system with two scanner units. The CG were asked to walk at two different speeds, at a self-selected speed and at a slower one, similar to the mean gait speed previously registered in the CCS patient group. Temporal, spatial variables and kinematic variables (maximum and minimum lower limb joint angles throughout the gait cycle in each plane, along with the gait cycle instants of occurrence and the joint range of motion - ROM) were compared between the two groups walking at similar speeds.

**Results:**

The kinematic parameters were compared when both groups walked at a similar speed, given that there was a significant difference in the self-selected speeds (p < 0.05). Hip abduction and knee flexion at initial contact, as well as minimal knee flexion at stance, were larger in the CCS group (p < 0.05). However, the range of knee and ankle motion in the sagittal plane was greater in the CG group (p < 0.05). The maximal ankle plantar-flexion values in stance phase and at toe off were larger in the CG (p < 0.05).

**Conclusions:**

The gait pattern of CCS patients showed a decrease of knee and ankle sagittal ROM during level walking and an increase in hip abduction to increase base of support. The findings of this study help to improve the understanding how CCS affects gait changes in the lower limbs.

## Background

Incomplete spinal cord injury (SCI), comprising about 30% of cases, is the most frequent form of SCI [1]. The central cord syndrome (CCS) is considered the most common incomplete SCI syndrome with a reported incidence varying from 15.7% to 25% [2]. CCS was first described by Schneider as a condition that is associated with sacral sparing and it is characterized by motor weakness that affects more the upper extremities than the lower limbs [3]. Independent ambulation was achieved in 87-97% in younger patients compared to 31-41% in patients older than 50 years at the time of injury [4].

The effect that the level of the lesion has on spasticty during walking has been studied in SCI patients [5], as have the changes in gait in patients with cervical myelopathy following therapeutic interventions [6], and even the gait of children and adolescents with SCI [7]. However, there are few studies that have focused on the biomechanics of gait in patients with CCS. To date, comparative biomechanical data has only been obtained in such patients for gait aided by one or two walking sticks [8]. However, the need to use biomechanical analyses to evaluate this patient group has been already emphasised [7,9]. The specific walking disorders occurring after incomplete SCI have been scarcely described in the literature. A recent study described the disturbances in the gait patterns of children and adolescents with SCI underscoring the importance of gait analysis as a tool to take therapeutic decisions, such as the prescription of orthosis or a surgical procedure, and to evaluate the patient during treatment or after surgical intervention [7].

Walking problems following CCS and other incomplete SCI syndromes have led to a wave of interest in using specific treatments, such as botulinum toxin type A [10] in combination with splinting to correct gait patterns. Different gait analyses have been carried out in several neuro-motor disorders [6,11,12]. These studies provide the basis to describe the type of gait disturbances that can be expected in these groups of patients and serve to define a rehabilitation therapy with realistic goals. In this context, the aim of the present study is to analyze the gait characteristics of subjects with CCS in order to quantify their gait pattern, and to compare these findings with a healthy age and sex matched control group using three-dimensional gait analysis walking at a self selected speed and at similar speed in both groups. The hypothesis tested was that kinematic values would in most cases be significantly different to those from a normal population, not only in the spatial-temporal parameters of gait but also in the joint motion. Accordingly, the findings obtained from the kinematic analysis of gait performed here should help to define the treatment necessary to resolve the problems detected.

## Methods

### Subjects

Twelve patients suffering from CCS participated in the experiments. Their average age was 42.6 ± 17.3 years (range, 21-61 years), height 162 ± 0.1 cm (range, 146-186 cm) and weight 68.7 ± 15.6 kg (range, 40-89 kg: Table 1). The inclusion criteria were:

**Table 1:** Clinical characteristics of both groups

Variable	CCS group (n = 12)	Control group (n = 20)
Sex (men)^†^	8 (67)	12 (60)
Age (years)*	42.58 (17.3)	34.50 (9.8)
Height (cm)*	162 (13.44)	167 (8.08)
Weight (kg) *	68.7 (15.6)	65.9 (10.8)
Time since injury (months)*	16.2 (15.7)	NA
Age when injury (years)*	40.5 (16.4)	NA
Level of injury C1^†^	1 (8.3)	NA
Level of injury C4^†^	5 (41.6)	NA
Level of injury C5^†^	2 (16.6)	NA
Level of injury C6^†^	2 (16.6)	NA
Level of injury C7^†^	2 (16.6)	NA
Right upper limb motor score(maximum 25)*	19.5 (3.1)	25
Left upper limb motor score (maximum 25)*	19.6 (3.5)	25
Right lower limb motor score (maximum 25)*	21.7 (3.2)	25
Left lower limb motor score (maximum 25)*	21.4 (3.9)	25
Upper Limb Motor Score (maximum 50)	33.83 (4.41)	50
Lower Limb Motor Score (maximum 50)	42.33 (5.19)	50
Average between upper limb and lower limb motor score.	8.50	NA
Ashworth score*	1.21 (0.2)	NA
WISCI II^†^	20 (100)	NA
TUG (seconds)*	17.1 (6.9)	NA
10MWT (seconds)*	17.4 (6.7)	NA

^†^Data are expressed as number (%) for categorical variables

*Data are expressed as mean (SD) for continuous variables

• Age range between 18 and 65 years.

• Clinical diagnosis of CCS: Patients with Spinal Cord Injury that displayed motor weakness affecting the upper limbs more than the lower limbs [3].

• Absence of previous history of locomotor or neurologic abnormality.

• Injury at least 12 months old.

The exclusion criteria were:

- Passive restriction of the joints.

- A diagnosis of any other neurological or orthopaedic disease that could affect locomotion.

- A diagnosis of any other disease associated with memory, concentration and/or visual deficits.

- Failure to comply with any of the criteria for inclusion.

Data from CCS patients were compared to an age, sex and anthropomorphically-matched healthy control group (CG) that included 20 subjects (12 male and 8 female). Their average age was 34.5 ± 9.8 years (range, 22-65), height, 167 ± 0.1 cm (range 157-184 cm) and weight 65.9 ± 10.8 kg (range 51-95 kg). All the participants provided informed consent prior to be included in this study and the study design was approved by local ethics committee.

### Materials

**Figure 1:**
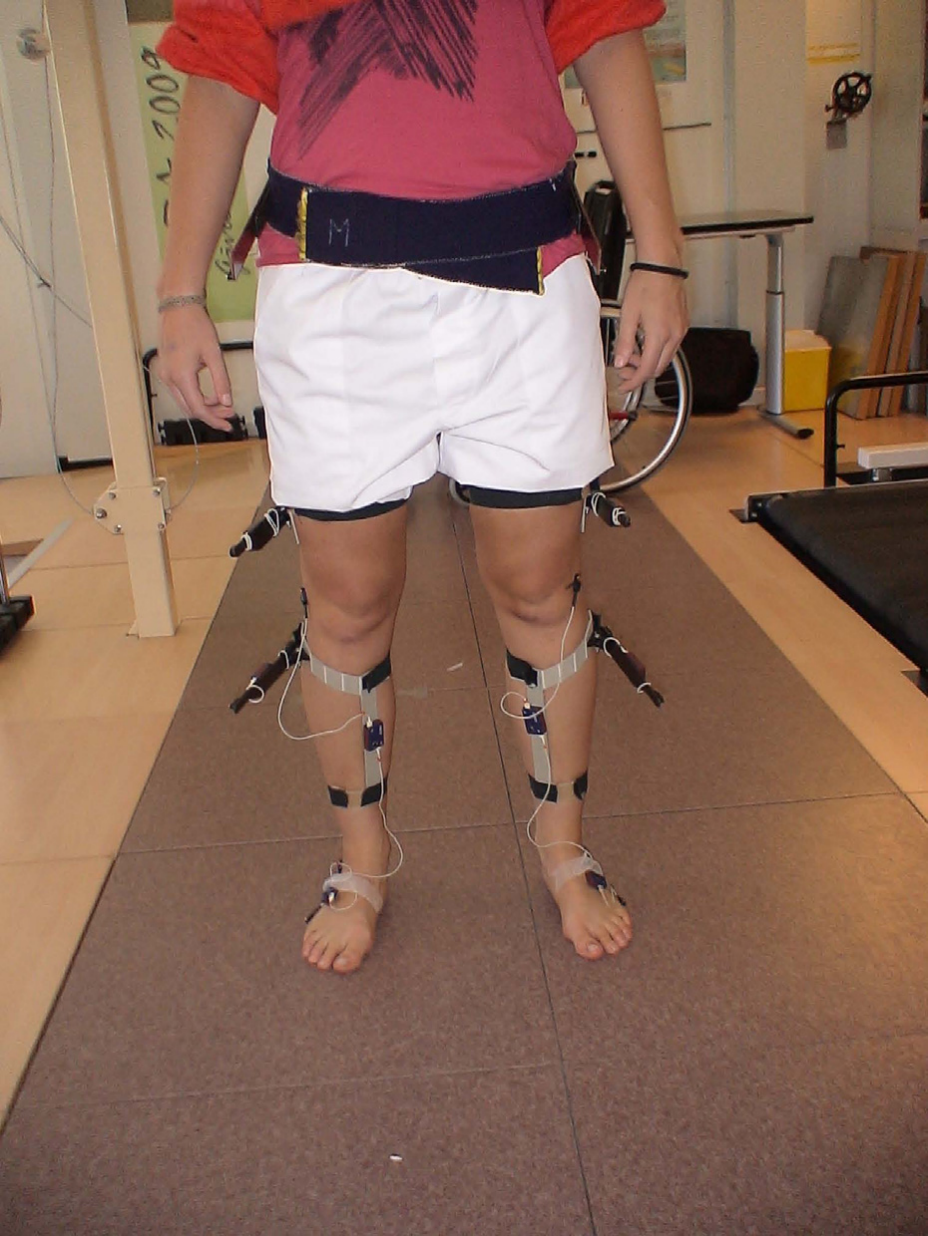
**Marker placement in a subject**. Frontal plane.

**Figure 2:**
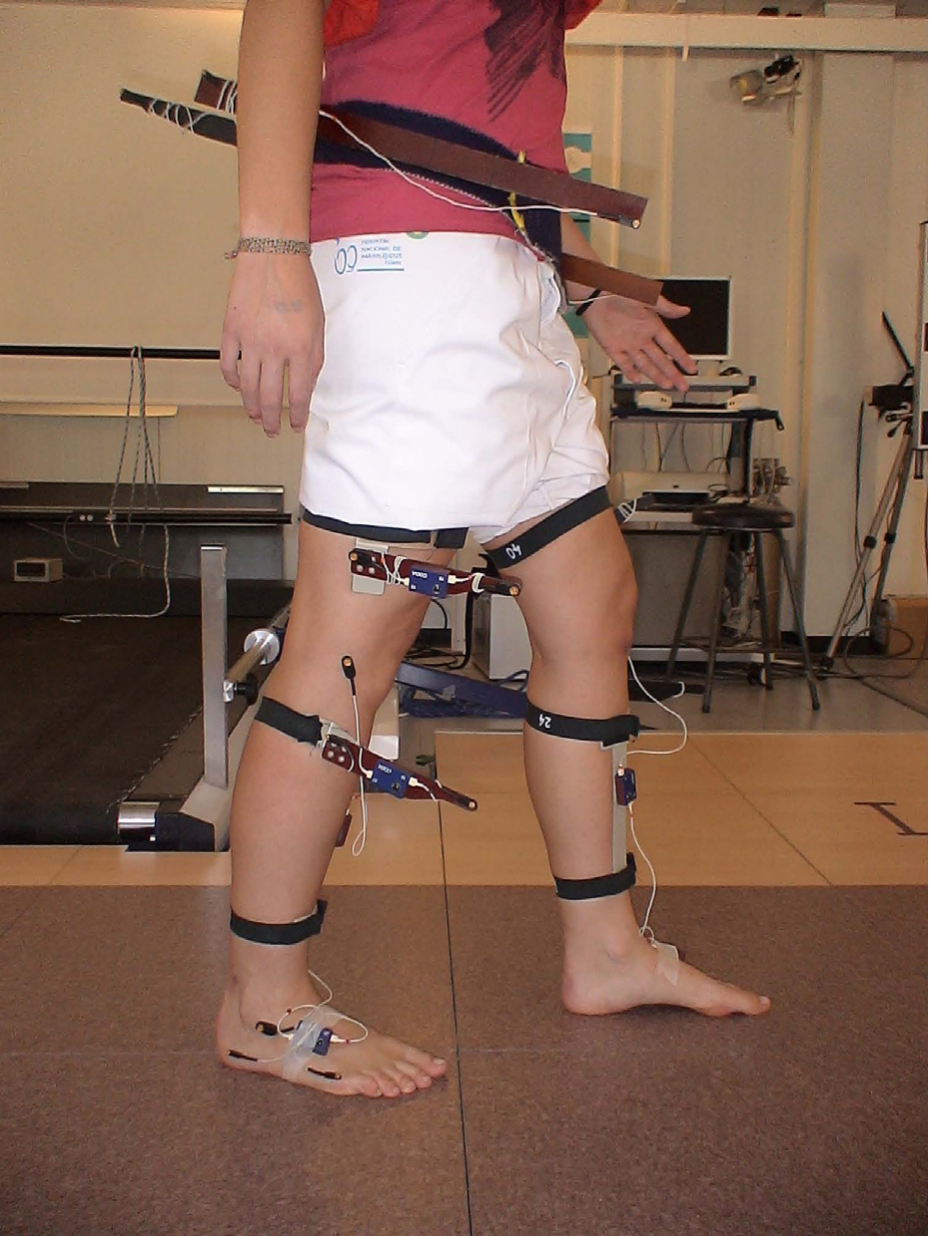
**Marker placement in a subject**. Sagital plane.

Kinematic data were recorded at 200 Hz using a three-dimensional motion analysis system (CODA System.6, Charnwood Dynamics, Ltd, UK) with two scanner units. Eleven active markers were placed on each lower limb (Figures 1 and 2) following a model described previously [8]. The recording was obtained simultaneously from both sides.

### Data collection

All CCS patients were asked to walk barefoot along a 10-m long walkway at a self-selected speed while temporal-spatial and kinematic data were recorded. It must be noted that all the kinematic parameters of gait depend on the speed [13]. Therefore, the CG were asked to walk at two different speeds, at a self-selected speed and at a slower one that was similar to the mean gait speed registered previously in the CCS patient group. Considering that the average speed of the patients was 0.7 m/s (SD = 0.2), the slow speed trials of the healthy controls were only included when the walking speed were between 0.7 m/s and 1.2 m/s [13]. The subjects in the control group were helped to walk more slowly with vocal commands.

Five valid trials were collected for each patient at a self selected speed and for CG at a self selected speed and at slow speed to reduce intrasubject variability. All the subjects were given a 1-minute rest period between trials.

### Data analysis

For each trial, a single gait cycle corresponding to the patient's cycle when crossing the midpoint of a 10-m walkway was selected to ensure that the gait pattern was free of the influence of the initial acceleration and the final braking. The temporal-spatial variables registered were: gait velocity, stride length, step length, stride time, step time, strides/minute, steps/minute or cadence, and percentage of stance phase duration. The joint motion data included: maximum and minimum value of lower limb joint angles throughout the gait cycle in 3 planes, along with the gait cycle instants of occurrence and the joint range of motion (ROM). Both groups of variables were compared between the two groups, CCS and CG.

The data from right and left limbs were averaged. All temporal events were expressed as gait cycle percentages (0-100%), defined between two consecutive heel-strikes of the same limb. The spatial parameters, speed, stride length, and step length were normalised by the subject height [5,11].

### Statistical analysis

For each subject, each computed parameter was calculated as the average of the values obtained in the five trials considered. A descriptive analysis was made of the clinical and functional variables by calculating the mean and standard deviation of the quantitative variables and the frequencies and percentages of the qualitative variables.

The normality distribution was checked for all the variables using the Kolmogorov-Smirnov test. Equality of variances was evaluated by Levene's test. Data were analysed using several one-way ANOVA tests (CCS group/CG group) with p = 0.05. All statistical analyses were performed using SPSS 12.0 (SPSS Inc, Chicago, IL, USA).

We certify that all applicable institutional and government regulations concerning the ethical use of human volunteers were followed during the course of this research.

## Results

### Clinical measurements

All patients had a cervical injury and they were classified as ASIA D [14]. The results of the clinical and functional assessment scales, such as Asworth score for spasticity measurement [15], WISCI (Walking Index Spinal Cord Injury) [16], TUG (Time Up and Go) [17] and 10MWT (10 Meter Walking Test) [17] most commonly used in this type of patient are shown in Table 1. The motor scores of both the upper limbs and lower limbs on both sides were similar, indicating symmetrical involvement [14], and the mean Ashworth score was 1.21 ± 0.2, which indicates that this group of patients does not suffer from pronounced spasticity [15]. None of the CCS patients needed a crutch to walk.

### Healthy control group at self selected speed versus patients with CCS

Significant differences between both groups were obtained in all of the temporal-spatial parameters when walking at self-selected speed (Table 2). Given these differences and that speed affects the kinematic parameters, possibly acting as a confounding factor, a comparison was made with the kinematic data obtained when the control subjects walked at a speed similar to that of the CCS patients. In this way, we were sure that the differences observed in the kinematic parameters were not due to the speed of walking.

**Table 2:** Temporal-spatial parameters between CCS group and control group.

	CCS group (n = 12)			Control group (self-selected speed) (n = 20)			Control group (slow speed) (n = 20)		
**Variable**	**Units**	**Mean**	**DS**	**Mean**	**DS**	**P value**	**Mean**	**DS**	**P value**

Speed	m/s	0.72	±0.25	1.28	±0.11	**0.000**	0.71	±0.08	0.835
Speed*	%height	43.22	±15.09	76.79	±8.66	**0.000**	42.10	±4.45	0.806
Stride Length*	%height	58.20	±11.67	80.24	±4.26	**0.000**	61.62	±4.35	0.345
Stride Time	s	1.44	±0.32	1.06	±0.08	**0.002**	1.48	±0.15	0.685
Strides/Minute		43.37	±8.31	57.28	±4.48	**0.000**	40.98	±3.69	0.363
Step Length*	%height	29.38	±6.35	40.38	±2.23	**0.000**	30.64	±2.20	0.519
Step Time	s	0.72	±0.16	0.53	±0.04	**0.002**	0.74	±0.07	0.726
Cadence	Steps/Minute	87.09	±16.26	114.22	±9.21	**0.000**	82.57	±7.44	0.380
Single Support	%cycle	0.44	±0.05	0.38	±0.02	**0.000**	0.45	±0.04	0.494
Double Support	%cycle	0.27	±0.13	0.15	±0.02	**0.006**	0.28	±0.05	0.874
Percentage stance	%cycle	68.41	±4.58	63.99	±1.19	**0.007**	69.20	±1.81	0.573

Significant difference between conditions at P < 0.05.

*Height-corrected values

### Healthy control group and patients with CCS at a matched speed

#### a) Temporal-spatial parameters

There were no significant differences in these parameters (Table 2).

#### b) Pelvis motion

Considering the average duration of the cycle, the maximal pelvic obliquity arose later in CCS patients than in controls, while the minimum obliquity occurred earlier in the patients. In addition, there was a slight anterior pelvic rotation in the CCS patients that was advanced in the gait cycle (Table 3).

**Table 3:** Pelvic kinematic parameters.

	CCS group (n = 12)			Control Group (slow speed) (n = 20)		
**Variable**	**Units**	**Mean**	**SD**	**Mean**	**SD**	**P value**

*PELVIS TILT*						

Maximum	degrees	20.26	±8.09	20.46	±4.39	0.939
Minimum	degrees	13.66	±7.371	15.14	±4.87	0.544
Range of motion	degrees	6.60	±2.48	5.32	±1.44	0.123
Time at max. pelvis tilt	% cycle	48.17	±10.50	38.52	±16.20	0.076
Time at min. pelvis tilt	% cycle	48.50	±12.32	57.16	±14.02	0.088

*PELVIS OBLIQUITY*						

Maximum	degrees	3.31	±1.62	3.79	±1.29	0.363
Minimum	degrees	-3.44	±1.64	-4.13	±1.31	0.200
Range of motion	degrees	6.75	±3.19	7.92	±2.56	0.265
Time at max. pelvis obliquity	% cycle	43.84	±23.85	26.79	±10.92	**0.010**
Time at min. pelvis obliquity	% cycle	51.91	±14.31	65.44	±16.05	**0.023**

*PELVIS ROTATION*						

Maximum	degrees	5.75	±2.07	4.66	±1.18	0.067
Minimum	degrees	-6.00	±2.49	-4.64	±1.28	**0.050**
Range of motion	degrees	11.74	±4.47	9.30	±2.28	**0.049**
Time at max. pelvis rotation	% cycle	29.74	±7.38	36.09	±5.71	**0.011**
Time at min. pelvis rotation	% cycle	64.98	±14.82	66.87	±13.72	0.716

Significant difference between conditions at P < 0.05.

#### c) Hip motion

**Figure 3:**
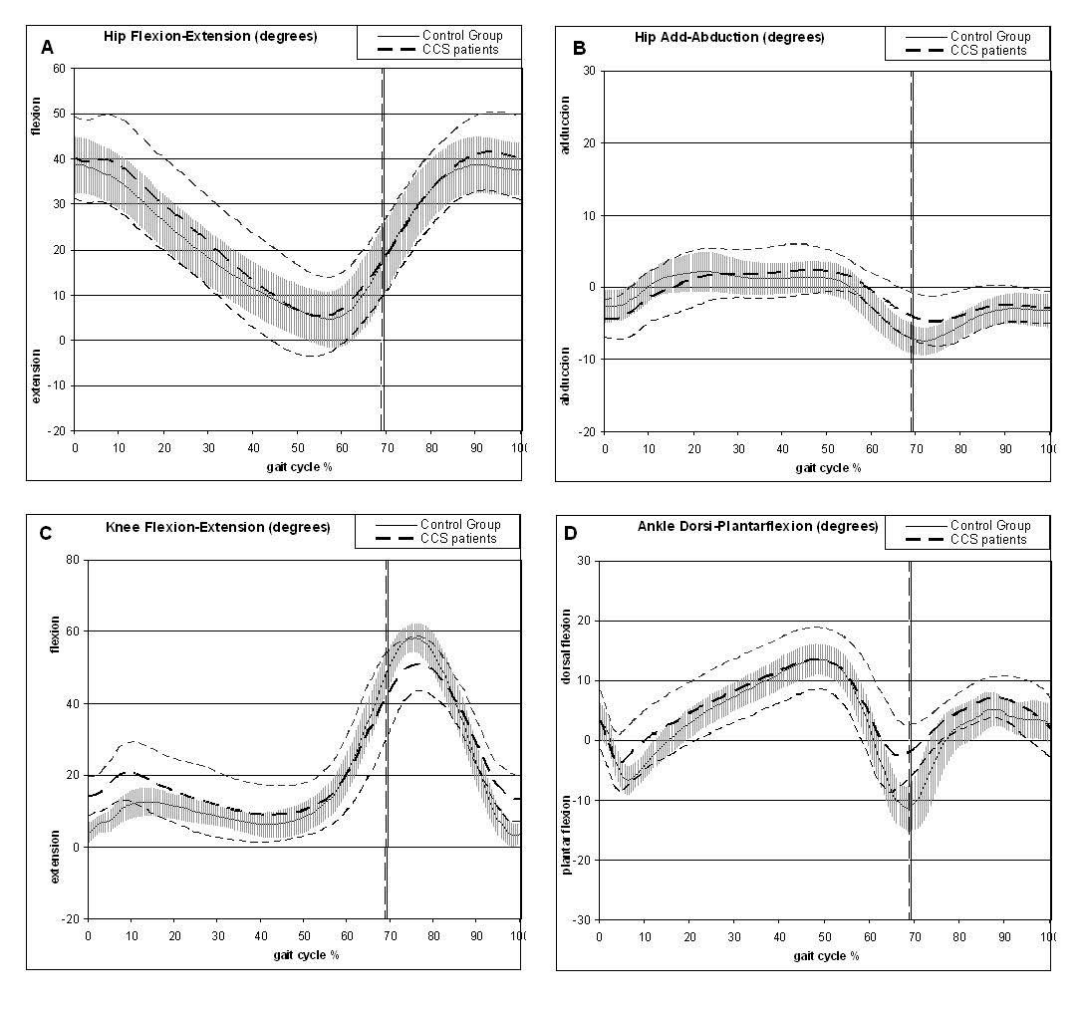
**Mean kinematic features of CCS patients (dashed line, mean and standard deviation) compared with the control group (continue thick line and grey line with standard deviation)**. The X-axis reflects the percentage of the gait cycle and on the Y-axis the units are in degrees. Kinematic curve for hip flexion-extension (A), hip adduction-abduction (B), knee flexion-extension (C) and the ankle dorsi-plantar flexion (D).

The maximal hip flexion during stance was significantly delayed in the group of CCS patients with respect to the control group (Figure 3a) and these differences were larger in the frontal plane (Table 4). At initial contact, the patients showed larger hip abduction, which reversed during the course of the stance phase as at toe-off, the control subjects showed larger hip abduction. Indeed, the CG subjects also had a larger hip abduction during swing (Table 4).

**Table 4:** Hip kinematic parameters

	CCS group (n = 12)			Control Group (slow speed) (n = 20)		
**Variable**	**Units**	**Mean**	**SD**	**Mean**	**SD**	**P value**

*HIP FLEXION-EXTENSION*						

Flexion at initial contact	degrees	40.20	±9.11	38.68	±6.41	0.584
Max. flex. in stance phase	degrees	41.24	±9.61	39.00	±6.28	0.430
Min. flex. in stance phase	degrees	4.14	±8.69	4.15	±6.41	0.998
Flexion at toe off	degrees	17.60	±10.17	17.92	±6.39	0.913
Max. flex. in swing phase	degrees	42.70	±8.79	39.45	±6.20	0.229
Min. flex. in swing phase	degrees	17.45	±9.96	17.92	±6.39	0.870
Range of motion	degrees	39.39	±6.27	36.26	±4.18	0.099
Time at max. flex. in stance phase	% cycle	4.57	±3.71	1.53	±1.65	**0.003**
Time at min. flex. in stance phase	% cycle	55.48	±2.82	57.17	±1.74	**0.044**
Time at flexion toe off	% cycle	68.41	±4.58	69.20	±1.81	0.573
Time at max. flex. in swing phase	% cycle	93.03	±2.71	92.90	±3.32	0.911
Time at min. flex. in swing phase	% cycle	68.84	±5.11	69.21	±1.81	0.813

*HIP ADDUCTIO-ABDUCTION*						

Abd. at initial contact	degrees	4.44	±2.61	2.56	±2.30	**0.041**
Max. add. in stance phase	degrees	3.99	±2.69	3.30	±2.32	0.451
Max. abd in stance phase	degrees	6.83	±2.77	7.63	±1.92	0.339
Adduction at toe off	degrees	-4.36	±3.61	-7.44	±2.05	**0.016**
Max. add in swing phase	degrees	-0.57	±2.54	-2.33	±2.12	**0.044**
Max. abd in swing phase	degrees	6.34	±2.84	7.99	±1.99	0.063
Range of motion	degrees	12.20	±3.25	11.42	±2.68	0.471
Time at max. add in stance phase	% cycle	41.94	±11.35	30.26	±11.97	**0.011**
Time at max. abd in stance phase	% cycle	35.06	±28.94	62.48	±10.44	**0.008**
Time at max. add in swing phase	% cycle	85.30	±10.23	92.34	±4.04	**0.040**
Time at max. abd in swing phase	% cycle	80.28	±7.85	72.36	±4.26	**0.006**

*HIP ROTATION*						

Maximum Internal rotation	degrees	1.29	±6.16	-0.486	±6.59	0.455
Minimum internal rotation	degrees	-12.17	±8.13	-12.58	±6.83	0.880
Range of motion	degrees	13.47	±4.63	12.09	±2.19	0.264
Time at max. internal rotation	% cycle	51.03	±14.98	49.77	±25.03	0.859
Time at min. internal rotation	% cycle	53.77	±26.00	59.82	±17.19	0.483

Significant difference between conditions at P < 0.05.

The maximal hip adduction during stance occurred earlier in the CG, while during swing the maximal hip adduction was delayed in the CG (Figure 3b). In fact, the maximal hip abduction values during stance were considerably delayed in the CG (Table 4).

#### d) Knee kinematics

The knee flexion at the initial contact was significantly greater in the patients although the maximal flexion during the stance phase was larger in the CG. However, the minimal knee flexion during swing and stance were larger in the CCS group, while knee flexion at toe off was lower in CCS. It must be noted that the CG reached a greater flexion during swing and they showed higher knee ROM in the sagittal plane (Table 5). In addition, the minimal knee flexion during swing was reached earlier in the CG (Figure 3c).

**Table 5:** Knee kinematic parameters.

	CCS group (n = 12)			Control Group (slow speed) (n = 20)		
**Variable**	**Units**	**Mean**	**SD**	**Mean**	**SD**	**P value**

*KNEE FLEXION*						

Flexion at initial contact	degrees	14.20	±5.50	4.03	±3.02	**0.000**
Max. flex. in stance phase	degrees	43.33	±8.91	48.72	±3.94	**0.025**
Min. flex. in stance phase	degrees	6.72	±6.60	2.87	±3.21	**0.034**
Flexion at toe off	degrees	44.25	±8.94	49.73	±3.92	**0.023**
Max. flex. in swing phase	degrees	53.53	±7.65	59.19	±3.76	**0,009**
Min. flex. in swing phase	degrees	12.67	±6.37	2.89	±3.44	**0.000**
Range of motion	degrees	47.51	±9.98	57.39	±4.37	**0.001**
Time at max. flex. in stance phase	% cycle	67.33	±6.30	68.86	±1.83	0.313
Time at min. flex. in stance phase	% cycle	30.38	±12.53	13.65	±12.76	**0.001**
Time at max. flex. in swing phase	% cycle	74.65	±3.15	75.26	±1.59	0.476
Time at min. flex. in swing phase	% cycle	98.76	±0.85	98.56	±1.10	0.601

*KNEE VARUS*						

Maximum	degrees	3.69	±3.62	5.06	±2.38	0.204
Minimum	degrees	-6.43	±6.68	-7.03	±4.20	0.757
Range of motion	degrees	10.13	±4.18	12.10	±3.98	0.193
Time at max. varus	degrees	59.92	±20.06	54.16	±19.61	0.431
Time at min. varus	degrees	56.91	±18.76	70.17	±9.22	**0.012**

*KNEE ROTATION*						

Maximum internal rotation	degrees	5.02	±5.79	4.47	±7.75	0.834
Minimum internal rotation	degrees	-8.56	±5.22	-9.52	±7.42	0.698
Range of motion	degrees	13.58	±2.83	13.99	±2.77	0.690
Time at max. internal rotation	% cycle	43.96	±20.79	43.57	±21.15	0.960
Time at min. internal rotation	% cycle	72.64	±13.10	73.85	±13.87	0.808

Significant difference between conditions at P < 0.05.

#### e) Ankle kinematics

The minimal dorsi-flexion or maximal ankle plantar-flexion during stance, at toe-off and during the swing phase was smaller in the CCS group. Consequently, the ankle flexo-extension ROM was higher in the CG.

The maximal value of the ankle plantar-flexion occurred earlier in the CCS patients during stance but not during swing (Figure 3d). Likewise, the instant of minimal supination occurred earlier in the CCS group (Table 6). However, the prono-supination ROM and the maximal supination values were higher in the CG.

**Table 6:** Ankle kinematic parameters.

	CCS group (n = 12)			Control Group (slow speed) (n = 20)		
**Variable**	**Units**	**Mean**	**SD**	**Mean**	**SD**	**P value**

*ANKLE DORSIFLEXION*						

Dorsiflexion at initial contact	degrees	3.29	±4.90	3.13	±3.15	0.912
Max. dorsi. in stance phase	degrees	15.07	±5.00	14.36	±2.62	0.600
Min. dorsi. in stance phase	degrees	-7.91	±4.98	-13.84	±3.66	**0.001**
Dorsiflexion at toe off	degrees	-4.05	±5.99	-12.98	±4.14	**0.000**
Max. dorsi. In swing phase	degrees	8.97	±3.75	6.72	±2.72	0.059
Min. dorsi. In swing phase	degrees	-4.99	±5.67	-13.15	±4.21	**0.000**
Range of motion	degrees	23.52	±6.10	28.50	±3.58	**0.007**
Time at max. dorsi. in stance phase	% cycle	47.98	±5.13	48.44	±2.97	0.749
Time at min. dorsi. in stance phase	% cycle	36.18	±20.75	62.63	±13.97	**0.000**
Time at max. dorsi. in swing phase	% cycle	87.54	±3.36	89.20	±4.34	0.265
Time at min. dorsi. in swing phase	% cycle	74.68	±10.09	69.43	±1.86	**0.029**

*ANKLE SUPINATION*						

Maximum	degrees	8.94	±9.77	15.55	±5.42	**0.019**
Minimum	degrees	-15.59	±7.79	-16.75	±11.68	0.761
Range of motion	degrees	24.53	±6.16	32.30	±11.66	**0.041**
Time at max. supination	degrees	68.07	±10.83	54.86	±21.26	0.055
Time at min. supination	degrees	52.00	±16.47	63.57	±11.64	**0.027**

Significant difference between conditions at P < 0.05.

## Discussion

The aim of this study was to objectively and quantitatively analyze and evaluate the gait of patients with CCS using three-dimensional kinematic movement analysis equipment, and to compare them with healthy subjects. This comparison was made at both a self-selected speed and at a matched speed in order to avoid any variation due to velocity. The main findings of this study should serve to define the basic rehabilitation strategies for CCS patients.

The results of our study reveal that not only do patients with CCS walk at a slower speed but also, that they display a series of kinematic alterations such as a smaller range of movement in the sagittal plane of the knee, greater abduction of the hip at the initial contact and during the oscillation phase, as well as a diminished range of joint movement in the ankle.

Some of these kinematic findings coincide with the data published elsewhere regarding the gait of patients with incomplete SCI [18,19], such as the limited flexion of the knee during the oscillation phase. Previously, the limited flexion of the knee during the oscillation phase was explained by the antagonistic action of the rectus femoris muscle and of the Vastus lateralis [18], leading to the recommendation that strategies are adopted to stretch these muscles or other such adaptations of clinical treatments to improve these patients' capacity to walk. This limited flexion in our group of patients was also evident, although we cannot confirm that it is due to the antagonistic action of the quadriceps since we did not register the electromyographic activity.

In our patients, the range of knee movement was diminished in the sagittal plane, whereby the knee was more flexed during the support phase and less flexed than in the control group during the oscillation phase. This reduced range of knee flexion has been observed in other studies of patients with paraplegic-spastic gait of diverse aetiology, in which this limitation was proposed to be correlated with the degree of spasticity [5]. The degree of spasticity is mild in our sample of patients, and they suffer no passive limitation to the joint movement. Accordingly, this alteration might be due to a specific loss muscle control, as suggested previously [20].

The reduced joint movement of the knee and ankle in the sagittal plane is not accompanied by a reduction in the hip, as seen elsewhere [6]. The normal peak of plantar flexion of the ankle is also diminished in patients with CCS and as occurs in other neurological disorders, this contributes to the reduced walking speed [21].

From a clinical point of view, the data obtained suggest that in patients with CCS, we should preferentially work on lengthening the ischiotibialis muscles and on muscle coordination to try to reduce the knee flexion at initial contact, and not only on strengthening the muscles. Indeed, while some studies indicate that an increase in strength in the lower limbs is related with an improvement in gait [22], others consider that this is not always the case [23].

Likewise, we also recommend stretching the anterior rectus femoris and the Vastus lateralis to help increase knee flexion during the oscillation phase and in general, to improve the range of knee mobility in the sagittal plane [18].

One issue that cannot be overlooked is the walking speed. It has been demonstrated that the speed at which we walk conditions the kinematic variables of our gait [13]. Our patients walk at a slower speed than the control group when walking at the self-selected speed, with shorter strides and a lower cadence, while the double support phase was longer. It has been reported that decreasing gait speed might be useful to prevent a fall when gait is perturbed [24,25].

These findings agree with earlier studies of patients with different neurological diseases such as patients with spastic paraplegia [26], cervical myelopathy [6] or Duchenne's muscular dystrophy [11]. For this reason, the subjects in the control group were also made to walk at a similar speed as the group of patients with CCS. For the control subjects to walk more slowly, they reduced the length of their stride and their cadence, and they increased the duration of the support phase, as demonstrated in previous studies [13]. In this way, we ensured that the speed did not influence the kinematic variables, although we must also bear in mind that this may introduce a certain bias in the data from the control group since walking slowly may modify their normal gait.

Since there are many parameters that can be obtained from gait analysis, it is necessary to take into account the reliability of measurements in different joint planes. In marker based gait analysis, some of these parameters can be obtained with greater precision (hip and knee ROM in the sagittal plane) than others (such as hip or knee rotation), since a larger movement is measured.

There are certain limitations associated with this study, the principal one being the lack of kinetic and electromyographic data. Since we are aware of the importance of such data, we have now introduced the necessary modifications to our equipment so that these parameters can be incorporated in future studies. Despite this limitation, the data regarding gait has been collected from the largest group of CCS patients yet studied. To date, the only study of CCS patients published using a three-dimensional analysis of movement to evaluate the kinematics of gait did not describe the pattern obtained in these patients but rather, it compared these CCS patients walking with the aid of one or two walking sticks to evaluate the improvement in this population [8]. Thus, there was no attempt to describe the kinematic differences with respect to a control group of subjects. Hence, we consider that our data represents the first attempt to define the alterations in joint movement associated with this type of disorder, which should help improve the strategies adopted in rehabilitation therapies.

We believe it is difficult to perform studies on this type of population given that there is still no clear consensus regarding the diagnostic criteria. However, a recent review concluded [27] established that the existence of a difference of at least 10 points between the motor index of upper and lower limbs served as a good diagnostic criterion for CCS [27]. In our cohort, the mean difference in the motor index of upper and lower limbs was 8.5 points. Although we are aware that this does not reach the minimum threshold of 10 points, the difference is small and as such, the results presented here are likely to be relevant. Nevertheless, the small difference in the motor index found leads us to assume that our group of patients suffer a mild form of CCS.

## Conclusion

CCS patients experience a decrease of knee and ankle sagittal motion during level walking and an increase of hip abduction. The reduction in the range of motion of these joints cannot be attributed to increased spasticity but rather to other compensatory mechanisms aimed at improving gait stability, and to the neural damage suffered by the patients.

The findings of this study help to improve the understanding how CCS affects gait changes in the lower limbs and how to design rehabilitation strategies for their treatment.

## Consent statement

Written informed consent was obtained from the patient for publication of this research and accompanying images. A copy of the written consent is available for review by the Editor-in chief of this journal.

## Competing interests

None of the authors of this paper have any conflict of interest in relation to any sources of any kind pertinent to this study. No commercial party having a direct financial interest in the results of the research supporting this article has or will confer a benefit upon the author(s) or upon any organization with which the author(s) is/are associated.

## Authors' contributions

AGA contributed to the concept and design, planning of study, analysis and interpretation of the data, drafting and completion of the manuscript. AFC contributed to design, analysis, completion of the manuscript and analysis of the data. EPR contributed to the concept, software development, design and acquisition of the data. SPN contributed to the analysis and acquisition of the data. BCR contributed to the analysis and acquisition of the data. AAE contributed to the software development, analysis and acquisition of the data. All authors read and approved the manuscript to be published.
